# A Text-Book of Pathology

**Published:** 1899-07

**Authors:** 


A Text-book of Pathology. By Alfred Stengel, M. D., Instructor in Clinical Medicine in the University of Pennsylvania; Professor of Clinical Medicine in the Woman's Medical College, Physician to the Philadelphia Hospital, Physician to the Children's Hospital, etc. With 372 illustrations. Octavo, pp. 848. Philadelphia: W. B. Saunders. 1898.

This work has been written from the standpoint of the clinical pathologist and considerable parts of it were first prepared and used as the basis of demonstrations upon clinical pathology for students of medicine. It has, therefore, grown from small beginnings and met the requirements of clinical teaching until it has developed into a large, handsome volume of 850 pages. The author has had abundant material to draw upon—both clinical and pathological—and as a student of the late lamented Dr. Pepper has had unexceled advantages and the best of counsel and example.

Dr. Stengel divides the work into two great parts. Part I. dealing with general pathology and Part II. with special pathology. Under general pathology are chapters dealing with the etiology of disease; disorders of nutrition and metabolism; disturbances of the circulation of the blood; retrogressive processes, as atrophy, hypertrophy, the infiltrations and degenerations; inflammation and regeneration: the tumors, bacteria and bacterial diseases. Animal parasites and diseases caused by them.

Under special pathology are treated, carefully and quite fully, the diseases of the blood and special organs. The author has drawn largely from works on pathology—special monographs in English, German and French—which coupled with his own extensive observation and experience has evolved a most creditable and valuable text-book on this most important subject. The illustrations, so important and invaluable in a work on pathology are beautiful, well-chosen and nicely executed. Some are in colors while others are half-tones.

A carefully prepared index is appended. The only feature that mars the all-around excellency of the book is a catalogue of books published by the makers of the book and bound into the book. Otherwise the publishers are to be complimented on their success in making the book-work comparable with the book contents.

